# Toll-Interacting Protein in Resolving and Non-Resolving Inflammation

**DOI:** 10.3389/fimmu.2017.00511

**Published:** 2017-05-05

**Authors:** Elizabeth J. A. Kowalski, Liwu Li

**Affiliations:** ^1^Department of Biological Sciences, Virginia Polytechnic State University, Blacksburg, VA, USA

**Keywords:** low-grade inflammation, toll-like receptor 4, toll-interacting protein, lipopolysaccharide, lysosome fusion, mitochondria

## Abstract

Innate leukocytes manifest dynamic and distinct inflammatory responses upon challenges with rising dosages of pathogen-associated molecular pattern molecules such as lipopolysaccharide (LPS). To differentiate signal strengths, innate leukocytes may utilize distinct intracellular signaling circuitries modulated by adaptor molecules. Toll-interacting protein (Tollip) is one of the critical adaptor molecules potentially playing key roles in modulating the dynamic adaptation of innate leukocytes to varying dosages of external stimulants. While Tollip may serve as a negative regulator of nuclear factor κ of activated B cells signaling pathway in cells challenged with higher dosages of LPS, it acts as a positive regulator for low-grade chronic inflammation in leukocytes programmed by subclinical low-dosages of LPS. This review aims to discuss recent progress in our understanding of complex innate leukocyte dynamics and its relevance in the pathogenesis of resolving versus non-resolving chronic inflammatory diseases.

## Current Knowledge of Low-Grade Inflammation and Limitations

The innate immune system plays a pivotal role in the immediate recognition of pathogen-associated molecular patterns (PAMPs) through pattern recognition receptors (PRRs) and the subsequent induction of the inflammatory responses (1). Upon PAMP recognition, cell surface PRRs will activate intracellular adaptor molecules, protein kinases, and transcription factors (2). These molecules will trigger the subsequent inflammatory responses. The stimulation of PRRs and the signal transduction pathways associated with them ultimately result in gene expression of cytokines, chemokines, cell adhesion molecules, and immune receptors (3). This broad range of molecules together coordinates the complex responses of the host to infection and other inflammatory stimulants.

Among the germ line-encoded PRRs, the toll-like receptors (TLRs) play an intricate role in innate immune system regulation and the inflammatory response. These TLRs recognize a wide range of PAMPs such as viral components and invariant bacterial components. TLR7, TLR8, TLR9, and TLR3 are located in the endolysosomal compartment and are responsible for detecting viral nucleic acids (4–8). By contrast, TLR2, TLR5, and toll-like receptor 4 (TLR4) detect different bacterial cell wall components and are localized on the cell surface. TLR7, TLR9, and TLR3 induce a robust type 1 interferon (IFN) response, which is a key for antiviral defense (9). TLR9 not only recognizes viral components but also uniquely recognizes bacterial deoxycytidylate-phosphate-deoxyguanylate (CpG)-DNA from bacteria and hemozoin from plasmodium and induces an IFN response (10, 11). Alternatively, TLR2, TLR5, and TLR4 may preferentially induce pro-inflammatory cytokines, although TLR4 ligand can be more pleiotropic and induce both inflammatory cytokines and IFN responses (12, 13). Given the intriguing complexity of TLR4 responses, we have been focusing on the dynamic modulation of TLR4 signaling networks. Lipopolysaccharide (LPS) is a ubiquitous surface component of Gram-negative bacteria and is recognized by innate immune cells through TLR4. It is well known that high dosages of bacterial endotoxin can induce a robust pro-inflammatory cytokine storm followed by a later refractory tolerant state with reduced cytokine expression (14). The cause of endotoxin tolerance is likely due to the induction of a multitude of negative regulators including IRAK-M, phosphatidylinositol-3-kinase (PI3K)/AKT, MKP-1, and SOCS (15).

However, an often-ignored effect of Gram-negative bacteria is low-grade non-resolving inflammation. Gram-negative bacteria occur naturally within the mucosal system, and shed endotoxin may permeate through leaky mucosal layers into circulation contributing to low-level endotoxemia (16, 17). In contrast to high doses of LPS, circulating concentrations of super-low-dose LPS (1–100 pg/mL) remain in humans with chronic infections, people with obesity, as well as individuals experiencing natural process of aging. It may also occur in individuals with life styles that include excessive drinking and chronic smoking (18–23). Low levels of endotoxin have been shown to cause persistent low-grade inflammation that is characterized by chronic low levels of pro-inflammatory mediators (24–29). Subclinical endotoxemia may program the host to a state of low-grade non-resolving inflammation, subjecting the host to more severe diseases (14, 30). Despite increasingly recognition that host innate leukocytes cannot only recognize the nature and identity but also the signal strengths of external stimulants, mechanisms responsible for the signal-strength-dependent leukocyte activation are not well understood.

## Molecular Circuitries Responsible for Signal-Strength-Dependent Innate Immunity Responses

As mentioned above, studies conducted with varying dosages of LPS led to the concept of innate immune programming dynamics and memory (31). Although extensive studies have revealed a large array of intracellular signaling molecules responsible for innate immune cell responses to LPS, their context-dependent modulations in response to varying dosages of LPS have just gotten attention. TLR4 is expressed on the surface of both hematopoietic and non-hematopoietic cells (32, 33). Like most surface receptors, TLR4 contains both an extracellular domain and an intracellular domain that has a highly regulated signaling cascade that follows activation. Though TLR4 is important for LPS recognition is has been shown that TLR4 alone may not be sufficient to elicit an inflammatory response. Myeloid differentiation factor 2 (MD2) must have physical association with TLR4 in order to induce ligand activation (34). In addition, together with lipid-binding protein, cluster of differentiation 14 (CD14) as well as CD11b also play critical roles in LPS sensing by TLR4. CD14 serves as a chaperone to recruit LPS to the TLR4–MD2 complex and is required for macropinocytosis in BMDM and DCs (35). CD11b may modulate LPS-induced signaling through both MyD88-dependent and -independent pathways (36). Through yet to be determined mechanisms, LPS interaction with the TLR4 complex may trigger the differential recruitment of downstream adaptor proteins such as toll/IL-1R homology, MyD88, TRAF, TIR-domain-containing adaptor protein inducing interferon-β (TRIF), and TRAM (37).

Through the engagement of TLR4 receptor and possibly other less-defined coreceptors, varying dosages of LPS may selectively activate distinct intracellular adaptor molecules such as TIRAP, TRAM, MyD88, TRIF, SARM, and toll-interacting protein (Tollip) (38), through poorly defined dynamics. MyD88 has been widely implicated in the robust responses of innate leukocytes to high doses of LPS (39). Recruitment of MyD88 stimulates the phosphorylation of IL-1R-associated kinases (IRAKs). The pathway will then signal and activate many downstream molecules, which in turn phosphorylate and activate mitogen-activated protein kinases and IκB kinase complex, which leads to the activation of key transcription factors, nuclear factor κ of activated B cells (NFκB), and AP-1, as well as robust expression of pro-inflammatory cytokines (37). NFκB activation also induces the expression of inhibitor molecules such as IRAK-M, Tollip, IκB, and SOCS. With particular interest, Tollip may inhibit TLR4 signaling by binding to IRAK-1 at resting state, thus reducing the cellular inflammatory response (40). MyD88 pathway may also activate PI3K pathway that further contribute to the induction of negative regulators of inflammatory processes (39, 41). Collectively, these negative regulators serve as negative feedback mechanisms to induce endotoxin tolerance.

By sharp contrast, super-low-dose LPS does not induce robust activation of NFκB, and only mildly induce low-grade inflammatory responses (42). Super-low-dose LPS also fails to induce the expression of negative regulators, thus allowing the non-resolving low-grade inflammation to persist (43). Under such non-resolving inflammatory process, our recent study reveals that MyD88 is not the primary adaptor molecule being utilized in the signaling process. Rather, TRAM/TRIF and Tollip may step in and serve to propagate the low-grade inflammatory process (44). Tollip-deficient macrophages have reduced expression of pro-inflammatory cytokines only when challenged with a super-low-dose LPS signal (44, 45). These findings suggest that Tollip serves as a positive signal to propagate low-grade inflammation. This is in contrast to the inhibitory effect of Tollip on high-dose LPS induced strong NFκB activation and cytokine storm.

## Tollip Structure and Subcellular Localization

**Figure 1:**
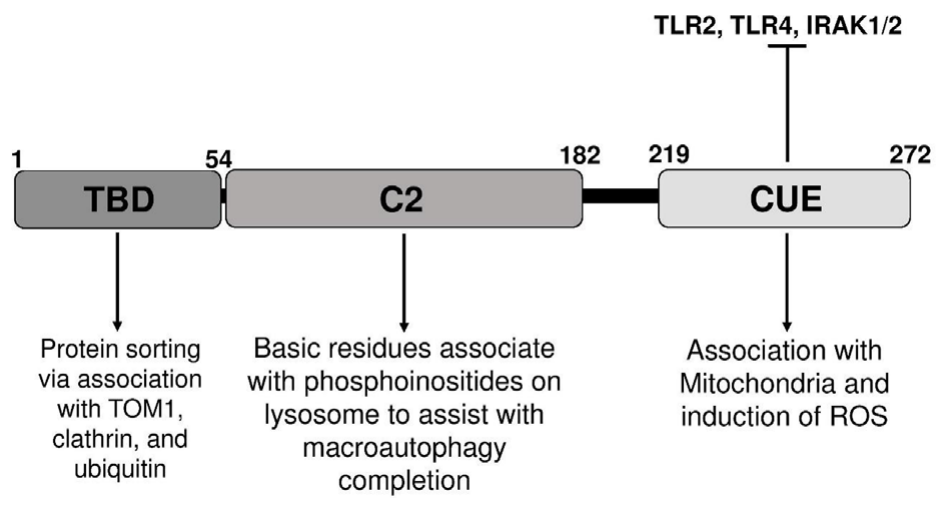
**Illustration of distinct domains of toll-interacting protein, relevant binding partners, and potential functions**.

**Figure 2:**
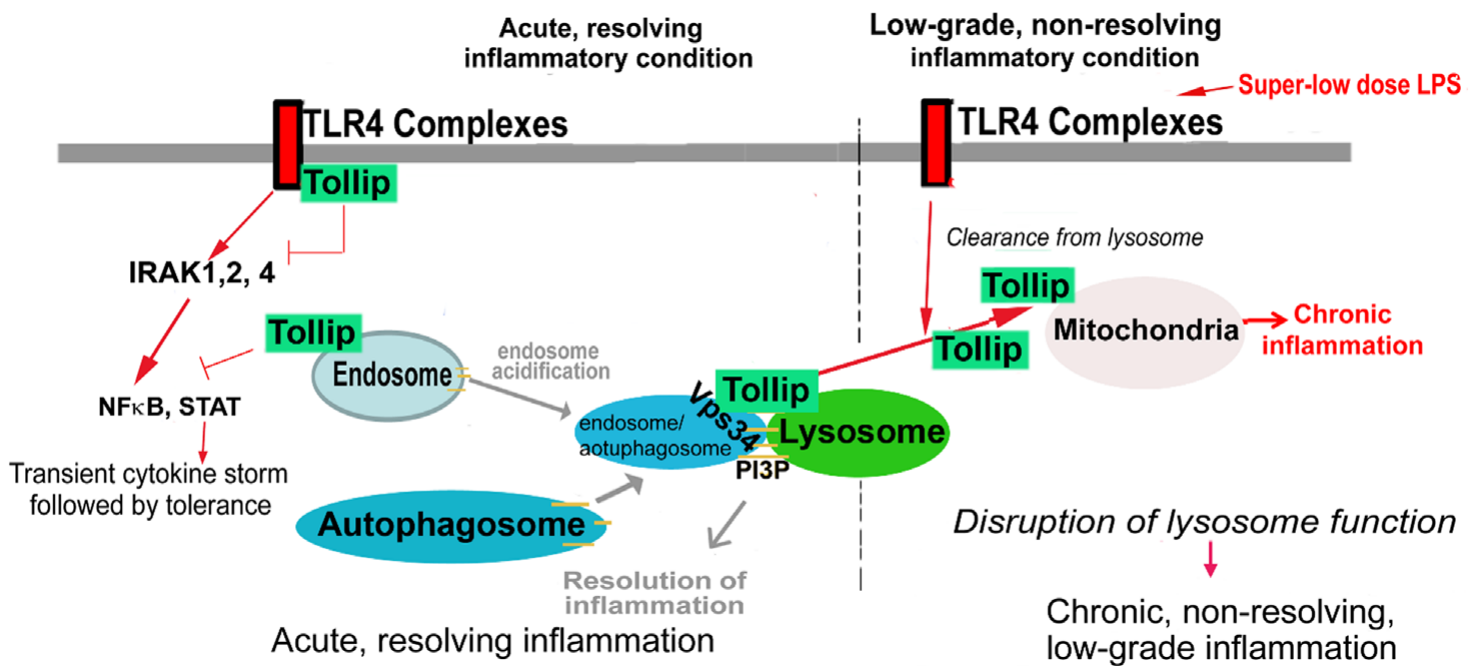
**Potential roles and modulations of toll-interacting protein (Tollip) in acute and chronic inflammation**. Under strong and acute inflammatory conditions, cell membrane-localized Tollip serves as a negative inhibitor for nuclear factor κ of activated B cells (NFκB) signaling pathway, facilitates the resolution of inflammation, and clearance of cellular debris as well as excessive lipids. However, under low-grade inflammatory conditions, Tollip undergoes translocation from cellular membrane to mitochondria. Dislocated Tollip loses its homeostatic function, fails to facilitate lysosome fusion. Instead, mitochondrial Tollip may facilitate non-resolving low-grade inflammation.

At the structural level, Tollip has three distinct domains with the Tom1-binding domain (TBD), the conserved 2 domain (C2), and the coupling of ubiquitin to ER degradation (CUE) domain as seen in Figure 1 (46). The Tollip TBD is involved in protein sorting *via* association with target of Myb protein (TOM1), clathrin, and ubiquitin during early endosomal interactions (47). The TBD was recently shown to be disordered in its native state, but upon binding to the Tom1 GAT domain the structure composed of the first 22 amino acids becomes better organized. The C2 domain is found in over 100 different proteins and is approximately 130 residues in size. The C2 domain has been shown to bind to phospholipids in both a calcium-dependent and -independent manner (48). In proteins, such as synaptotagmin, calcium binding will not induce a confirmation change, but will affect the electrostatic potential that augment phospholipid binding (48). This suggests that the C2 domain functions primarily through electrostatic activation. As previously discussed, the C2 domain of Tollip has been shown to bind specifically to phospholipids and shows a broad preference for phosphoinositides, thus enabling Tollip localization with cellular membranes rich in phospholipid such as cell membrane, endosome, and lysosome (49–52). The ability of Tollip C2 domain to interact with PI(3)P was recently shown to be drastically diminished when the Tollip TBD binds with the Tom1 GAT domain. This study reveals that Tollip association with Tom1 may affect the PI(3)P binding of Tollip as well as its localization to endosome/lysosome (53, 54). The CUE domain is typically a much smaller domain of approximately 40 residues and performs a variety of functions, such as protein sorting and interacting with ubiquitinated proteins. The CUE domain is very similar to the ubiquitin-binding UBA domain, which contains a three-helix bundle. The CUE domain contains a conserved MFP and LL motif in the α-helix1 and α-helix3, respectively (55). These two motifs are well known for interacting with the hydrophobic patch of ubiquitin (55). When stimulated with high doses of LPS, Tollip may aggregate at cellular and/or lysosome membranes with IL-R1 and TLR4, contributing to the inhibition of TLR4-mediated immune response *via* the CUE domain. Tollip also negatively regulates IRAK-1 and IRAK-2 by directly binding to these proteins *via* the CUE domain and inhibiting auto-phosphorylation (40) (Figure 2).

On the other hand, Tollip translocate to mitochondria in cells challenged with super-low-dose LPS (52). When the CUE domain is mutated at its MFP motif, causing an inability to interact with ubiquitinated proteins, the Tollip CUE domain mutant fails to translocate to mitochondria and remains at endosome–lysosome (52). The molecular mechanisms for Tollip translocation are not clear. Structural analyses suggest that ubiquitin binding *via* Tollip CUE domain may reduce its interaction with phospholipids (56). Since phospholipids are primarily localized at cell membrane, endosome, lysosome, and Golgi, but not on mitochondria (57), enhanced ubiquitin interaction and reduced phospholipid binding of Tollip may be responsible for its translocation of Tollip from lysosome membrane to mitochondria. These molecular and cellular studies suggest that Tollip may play distinct roles in modulating inflammation through its differential subcellular localization.

## Lysosomal Tollip and Its Potential Role in Resolution of Inflammation

Toll-interacting protein may associate with cell membrane and/or other intracellular membrane structures such as endosomes, lysosomes, and Golgi, due to its affinity with phosphoinositide through its C2 domain. Tollip has been shown through kinetic studies to reversibly bind to PtdIns3*P* (phosphatidylinositol 3-phosphate) and PtdIns(4,5)*P*_2_ (phosphatidylinositol 4,5-bisphosphate), with low micromolar affinity (51). Through its phospholipid interaction, Tollip may fulfill its homeostatic role by inhibiting IRAK-mediated robust NFκB signaling and cytokine storm under acute and severe inflammatory conditions (58). Indeed, Tollip was shown to be critically important during the development of endotoxin tolerance, by suppressing the robust NFκB pathway and preventing cytokine storm (58). Tollip-deficient cells or mice fail to develop endotoxin tolerance when challenged with higher dose LPS (59).

Furthermore, PtdIns(4,5)*P*_2_ has been shown to be necessary for vacuole fusion and it has been speculated that PtdIns(4,5)*P*_2_ plays a direct role in membrane fusion by binding and recruiting specific molecules to the vacuoles being fused (60). PtdIns3*P* has also been shown to play an important modulatory role in autophagy (61). By interacting with these lipids, Tollip may fulfill its role in the fusion of the endosome/autophagosome with the lysosome (52). Proper fusion of autolysosome may enable efficient clearance of cellular stress molecules and restore cellular homeostasis (62). Lysosomes are not only critical for autophagy completion but also serve as major signaling platforms for innate immunity signaling by recruiting key signaling molecules such as MAVs and STATs (63). Tollip may serve as a negative regulator to dampen innate stress signaling processes at the lysosome platform (Figure 1).

## Mitochondrial Tollip and Its Role in Low-Grade Inflammation

Under low-grade inflammatory conditions, however, Tollip was shown to be cleared away from lysosome, thus compromising its homeostatic function (52). By sharp contrast, Tollip translocates to mitochondria through its CUE domain interaction upon stimulation with super-low doses of LPS. Mitochondrial Tollip, instead, is an important facilitator for the generation of mitochondrial reactive oxygen species (ROS), which drives the expression of pro-inflammatory mediators through the activation of selected transcription factors such as C/EBPδ (42, 64). Tollip-deficient macrophages have been shown to be unable to induce mitochondrial ROS (43). Along with ROS reduction in Tollip-deficient cells, there have also been reports of significantly decreased interleukin-6 and tumor necrosis factor α (TNFα) in Tollip-deficient cells (43, 45). Under low-grade chronic inflammatory conditions, Tollip-deficient mice have reduced levels of pro-inflammatory cytokines such as TNFα, interleukin-12, and elevated levels of anti-inflammatory cytokines such as transforming growth factor β (TGFβ) (65). Potentially due to its translocation away from lysosome, Tollip-deficient cells also express higher levels of IFN-induced genes. Together, these studies suggest that the mitochondria localization of Tollip may play an important role in the low-grade inflammatory response of innate leukocytes.

## Tollip Involvement in Disease

Translational studies with both animal models and human clinical studies in the recent years have yielded compelling data that support the role of Tollip in inflammatory diseases. For example, Tollip expression has been found to be significantly increased in ischemia–reperfusion (I/R)-challenged brain tissue of humans, rats, and mice *in vivo* (66). In this study, it was also discovered that Tollip-deficient mice are protected against acute I/R injury by reducing neuronal apoptosis through decreased expression of pro-inflammatory and pro-apoptotic genes, while increasing anti-apoptotic genes (66).

Recent genetic and mechanistic studies also reveal that Tollip is involved in the pathogenesis of gut mucosal inflammatory syndromes such as inflammatory bowel disease (IBD), Crohn’s disease (CD), and ulcerative colitis (UC) (59, 67–69). These syndromes may be results of altered microbiome as well as altered mucosal immune environment. TLR4 expression is significantly increased in IBD, while Tollip expression is significantly decreased in both active and inactive UC and CD (59, 69). We recently reported that Tollip-deficient mice suffer from more severe chemically induced acute colitis with unabated expression of pro-inflammatory cytokines (59).

Genetic variants in human *TOLLIP* gene have been associated with idiopathic pulmonary fibrosis (IPF) (70). IPF is a devastating disease and is characterized by an interstitial fibrotic process and high mortality, which has an unknown etiology. Although lung transplant may hold treatment potential, the immunosuppression associated with transplant therapies may cause severe side effects (71). There were three *TOLLIP* single nucleotide polymorphisms (rs111521887, rs5743894, rs5743890) identified in the genome-wide association study that were associated with protection against IPF. TOLLIP expression was decreased by 20% in patients carrying the rs5743890 allele. This allele showed protection from development of IPF, but once IPF was developed the patients had higher mortality rates (70). The other two variants, rs111521887 and rs5743894, showed decreased TOLLIP expression by 40 and 50%, respectively. Another SNP in *TOLLIP*, rs3750920, was also linked with IPF (72). This SNP was associated with decreased Tollip expression. Cell samples from patients with the rs3750920 SNP exhibit reduced TLR4 signaling activation when challenged with *N*-acetylcysteine (72). In addition to IPF, genetic variants in human *TOLLIP*, rs3168046 and rs3793965, have been associated with lung transplant primary graft dysfunction (PGD). These mutations increase the risk of PGD, which once again exemplifies the importance of Tollip in the regulation of inflammation and disease (73). It is likely that similar to IPF, decreased Tollip levels may contribute to increased expression of pro-inflammatory cytokines and subsequent graft rejection.

In a recent study from our group, we observed that Tollip-deficient mice tend to develop larger yet stable atherosclerotic plaques with increased lipid deposition as well as increased plaque content of smooth muscle cells and collagen (65). We reported that the increased lipid deposition may be due to disrupted lysosome fusion and compromised lipophagy due to Tollip deficiency (65). On the other hand, Tollip-deficient mice have reduced circulating levels of pro-inflammatory cytokines such as TNFα, and increased levels of anti-inflammatory TGFβ. This may explain the stable atherosclerosis phenotype with increased smooth muscle cells and collagen. Together, these data reveal compound phenotypes associate with Tollip variants and deficiency, and further suggest that Tollip may play pleiotropic roles in a context-dependent fashion as we discussed in this review.

## Author Contributions

All authors listed have made substantial, direct, and intellectual contribution to the work and approved it for publication.

## Conflict of Interest Statement

The authors declare that the research was conducted in the absence of any commercial or financial relationships that could be construed as a potential conflict of interest.

## Abbreviations

C2, conserved 2 domain; CD, Crohn’s disease; CD14, cluster of differentiation 14; CUE, coupling of ubiquitin to ER degradation domain; IFN, interferon; IL-6, interleukin-6; IL-12, interleukin-12; I/R, ischemia–reperfusion; IRAK, interleukin-receptor-associated kinase; LPS, lipopolysaccharide; MAPK, mitogen-activated protein kinase; MD2, myeloid differentiation factor 2; NFκB, nuclear factor κ of activated B cells; PI3K, phosphatidylinositol-3-kinase; ROS, reactive oxygen species; TBD, Tom1-binding domain; TIR, toll/IL-1R homology; TGFβ, transforming growth factor β; TNFα, tumor necrosis factor α; Tollip, toll-interacting protein; TRAF6, tumor-necrosis-factor-receptor-associated factor 6; TRIF, TIR-domain-containing adaptor protein inducing interferon-β; UC, ulcerative colitis.

## References

[1] Medzhitov R Janeway C Jr Innate immune recognition: mechanisms and pathways. Immunol Rev (2000) 173:89–97. 10.1034/j.1600-065X.2000.917309.x 10719670

[2] Akira S Takeda K Toll-like receptor signalling. Nat Rev Immunol (2004) 4(7):499–511. 10.1038/nri1391 15229469

[3] Akira S Uematsu S Takeuchi O Pathogen recognition and innate immunity. Cell (2006) 124(4):783–801. 10.1016/j.cell.2006.02.015 16497588

[4] Alexopoulou L Holt AC Medzhitov R Flavell RA Recognition of double-stranded RNA and activation of NF-kappaB by toll-like receptor 3. Nature (2001) 413(6857):732–8. 10.1038/35099560 11607032

[5] Diebold SS Kaisho T Hemmi H Akira S Reis e Sousa C. Innate antiviral responses by means of TLR7-mediated recognition of single-stranded RNA. Science (2004) 303(5663):1529–31. 10.1126/science.1093616 14976261

[6] Lund JM Alexopoulou L Sato A Karow M Adams NC Gale NW Recognition of single-stranded RNA viruses by toll-like receptor 7. Proc Natl Acad Sci U S A (2004) 101(15):5598–603. 10.1073/pnas.0400937101 15034168 PMC397437

[7] Lamphier MS Sirois CM Verma A Golenbock DT Latz E TLR9 and the recognition of self and non-self nucleic acids. Ann N Y Acad Sci (2006) 1082:31–43. 10.1196/annals.1348.005 17145922

[8] Beutler BA TLRs and innate immunity. Blood (2009) 113(7):1399–407. 10.1182/blood-2008-07-019307 18757776 PMC2644070

[9] Kawai T Akira S Antiviral signaling through pattern recognition receptors. J Biochem (2007) 141(2):137–45. 10.1093/jb/mvm032 17190786

[10] Bauer S Kirschning CJ Hacker H Redecke V Hausmann S Akira S Human TLR9 confers responsiveness to bacterial DNA via species-specific CpG motif recognition. Proc Natl Acad Sci U S A (2001) 98(16):9237–42. 10.1073/pnas.161293498 11470918 PMC55404

[11] Coban C Ishii KJ Kawai T Hemmi H Sato S Uematsu S Toll-like receptor 9 mediates innate immune activation by the malaria pigment hemozoin. J Exp Med (2005) 201(1):19–25. 10.1084/jem.20041836 15630134 PMC2212757

[12] Hayashi F Smith KD Ozinsky A Hawn TR Yi EC Goodlett DR The innate immune response to bacterial flagellin is mediated by toll-like receptor 5. Nature (2001) 410(6832):1099–103. 10.1038/35074106 11323673

[13] Toshchakov V Jones BW Perera PY Thomas K Cody MJ Zhang S TLR4, but not TLR2, mediates IFN-beta-induced STAT1alpha/beta-dependent gene expression in macrophages. Nat Immunol (2002) 3(4):392–8. 10.1038/ni774 11896392

[14] Morris MC Gilliam EA Li L Innate immune programing by endotoxin and its pathological consequences. Front Immunol (2014) 5:680 10.3389/fimmu.2014.00680 25610440 PMC4285116

[15] Morris MC Gilliam EA Button J Li L Dynamic modulation of innate immune response by varying dosages of lipopolysaccharide (LPS) in human monocytic cells. J Biol Chem (2014) 289(31):21584–90. 10.1074/jbc.M114.583518 24970893 PMC4118118

[16] Franceschi C Bonafe M Valensin S Olivieri F De Luca M Ottaviani E Inflamm-aging. An evolutionary perspective on immunosenescence. Ann N Y Acad Sci (2000) 908:244–54. 10.1111/j.1749-6632.2000.tb06651.x 10911963

[17] Castro AM Macedo-de la Concha LE Pantoja-Meléndez CA Low-grade inflammation and its relation to obesity and chronic degenerative diseases. Rev Med Hosp Gen Mex (2016). 10.1016/j.hgmx.2016.06.011

[18] Goto T Edén S Nordenstam G Sundh V Svanborg-Edén C Mattsby-Baltzer I. Endotoxin levels in sera of elderly individuals. Clin Diagn Lab Immunol (1994) 1(6):684–8. 8556521 10.1128/cdli.1.6.684-688.1994 PMC368391

[19] Wiedermann CJ Kiechl S Dunzendorfer S Schratzberger P Egger G Oberhollenzer F Association of endotoxemia with carotid atherosclerosis and cardiovascular disease: prospective results from the Bruneck Study. J Am Coll Cardiol (1999) 34(7):1975–81. 10.1016/S0735-1097(99)00448-9 10588212

[20] Cani PD Bibiloni R Knauf C Waget A Neyrinck AM Delzenne NM Changes in gut microbiota control metabolic endotoxemia-induced inflammation in high-fat diet-induced obesity and diabetes in mice. Diabetes (2008) 57(6):1470–81. 10.2337/db07-1403 18305141

[21] Szeto CC Kwan BC Chow KM Lai KB Chung KY Leung CB Endotoxemia is related to systemic inflammation and atherosclerosis in peritoneal dialysis patients. Clin J Am Soc Nephrol (2008) 3(2):431–6. 10.2215/cjn.03600807 18256376 PMC2390956

[22] Rao R Endotoxemia and gut barrier dysfunction in alcoholic liver disease. Hepatology (2009) 50(2):638–44. 10.1002/hep.23009 19575462 PMC6209509

[23] Lira FS Rosa JC Pimentel GD Souza HA Caperuto EC Carnevali LC Endotoxin levels correlate positively with a sedentary lifestyle and negatively with highly trained subjects. Lipids Health Dis (2010) 9(1):82. 10.1186/1476-511x-9-82 20684772 PMC2922209

[24] Maachi M Pieroni L Bruckert E Jardel C Fellahi S Hainque B Systemic low-grade inflammation is related to both circulating and adipose tissue TNFalpha, leptin and IL-6 levels in obese women. Int J Obes Relat Metab Disord (2004) 28(8):993–7. 10.1038/sj.ijo.0802718 15211360

[25] Manco M Putignani L Bottazzo GF. Gut microbiota, lipopolysaccharides, and innate immunity in the pathogenesis of obesity and cardiovascular risk. Endocr Rev (2010) 31(6):817–44. 10.1210/er.2009-0030 20592272

[26] Mehta NN McGillicuddy FC Anderson PD Hinkle CC Shah R Pruscino L Experimental endotoxemia induces adipose inflammation and insulin resistance in humans. Diabetes (2010) 59(1):172–81. 10.2337/db09-0367 19794059 PMC2797919

[27] Sun L Yu Z Ye X Zou S Li H Yu D A marker of endotoxemia is associated with obesity and related metabolic disorders in apparently healthy Chinese. Diabetes Care (2010) 33(9):1925–32. 10.2337/dc10-0340 20530747 PMC2928335

[28] Terawaki H Yokoyama K Yamada Y Maruyama Y Iida R Hanaoka K Low-grade endotoxemia contributes to chronic inflammation in hemodialysis patients: examination with a novel lipopolysaccharide detection method. Ther Apher Dial (2010) 14(5):477–82. 10.1111/j.1744-9987.2010.00815.x 21175546

[29] Laugerette F Vors C Geloen A Chauvin MA Soulage C Lambert-Porcheron S Emulsified lipids increase endotoxemia: possible role in early postprandial low-grade inflammation. J Nutr Biochem (2011) 22(1):53–9. 10.1016/j.jnutbio.2009.11.011 20303729

[30] Chaudhry H Zhou J Zhong YIN Ali MM McGuire F Nagarkatti PS Role of cytokines as a double-edged sword in sepsis. In Vivo (2013) 27(6):669–84. 24292568 PMC4378830

[31] Yuan R Li L. Dynamic modulation of innate immunity programming and memory. Sci China Life Sci (2016) 59(1):38–43. 10.1007/s11427-015-4998-x 26740103

[32] Kurt-Jones EA Sandor F Ortiz Y Bowen GN Counter SL Wang TC Use of murine embryonic fibroblasts to define toll-like receptor activation and specificity. J Endotoxin Res (2004) 10(6):419–24. 10.1179/096805104225006516 15588425

[33] Taylor KR Trowbridge JM Rudisill JA Termeer CC Simon JC Gallo RL. Hyaluronan fragments stimulate endothelial recognition of injury through TLR4. J Biol Chem (2004) 279(17):17079–84. 10.1074/jbc.M310859200 14764599

[34] Nagai Y Akashi S Nagafuku M Ogata M Iwakura Y Akira S Essential role of MD-2 in LPS responsiveness and TLR4 distribution. Nat Immunol (2002) 3(7):667–72. 10.1038/ni809 12055629

[35] Moore KJ Andersson LP Ingalls RR Monks BG Li R Arnaout MA Divergent response to LPS and bacteria in CD14-deficient murine macrophages. J Immunol (2000) 165(8):4272–80. 10.4049/jimmunol.165.8.4272 11035061

[36] Ling GS Bennett J Woollard KJ Szajna M Fossati-Jimack L Taylor PR Integrin CD11b positively regulates TLR4-induced signalling pathways in dendritic cells but not in macrophages. Nat Commun (2014) 5:3039. 10.1038/ncomms4039 24423728 PMC3905776

[37] Barton GM Medzhitov R. Toll-like receptor signaling pathways. Science (2003) 300(5625):1524–5. 10.1126/science.1085536 12791976

[38] Kawai T Akira S TLR signaling. Semin Immunol (2007) 19(1):24–32. 10.1016/j.smim.2006.12.004 17275323

[39] Laird MH Rhee SH Perkins DJ Medvedev AE Piao W Fenton MJ TLR4/MyD88/PI3K interactions regulate TLR4 signaling. J Leukoc Biol (2009) 85(6):966–77. 10.1189/jlb.1208763 19289601 PMC2698589

[40] Zhang G Ghosh S. Negative regulation of toll-like receptor-mediated signaling by Tollip. J Biol Chem (2002) 277(9):7059–65. 10.1074/jbc.M109537200 11751856

[41] Medina EA Morris IR Berton MT. Phosphatidylinositol 3-kinase activation attenuates the TLR2-mediated macrophage proinflammatory cytokine response to *Francisella tularensis* live vaccine strain. J Immunol (2010) 185(12):7562–72. 10.4049/jimmunol.0903790 21098227

[42] Maitra U Gan L Chang S Li L Low-dose endotoxin induces inflammation by selectively removing nuclear receptors and activating CCAAT/enhancer-binding protein delta. J Immunol (2011) 186(7):4467–73. 10.4049/jimmunol.1003300 21357541

[43] Maitra U Deng H Glaros T Baker B Capelluto DGS Li Z Molecular mechanisms responsible for the selective and low-grade induction of pro-inflammatory mediators in murine macrophages by lipopolysaccharide. J Immunol (2012) 189(2):1014–23. 10.4049/jimmunol.1200857 22706082 PMC3392521

[44] Yuan R Geng S Li L. Molecular mechanisms that underlie the dynamic adaptation of innate monocyte memory to varying stimulant strength of TLR ligands. Front Immunol (2016) 7:497. 10.3389/fimmu.2016.00497 27891130 PMC5103159

[45] Didierlaurent A Brissoni B Velin D Aebi N Tardivel A Käslin E Tollip regulates proinflammatory responses to interleukin-1 and lipopolysaccharide. Mol Cell Biol (2006) 26(3):735–42. 10.1128/MCB.26.3.735-742.2006 16428431 PMC1347014

[46] Capelluto DG. Tollip: a multitasking protein in innate immunity and protein trafficking. Microbes Infect (2012) 14(2):140–7. 10.1016/j.micinf.2011.08.018 21930231

[47] Yamakami M Yoshimori T Yokosawa H. Tom1, a VHS domain-containing protein, interacts with Tollip, ubiquitin, and clathrin. J Biol Chem (2003) 278(52):52865–72. 10.1074/jbc.M306740200 14563850

[48] Sutton RB Davletov BA Berghuis AM Sudhof TC Sprang SR. Structure of the first C2 domain of synaptotagmin I: a novel Ca2+/phospholipid-binding fold. Cell (1995) 80(6):929–38. 10.1016/0092-8674(95)90296-1 7697723

[49] Li L Hu J Li L. Characterization of Tollip protein upon lipopolysaccharide challenge. Mol Immunol (2004) 41(1):85–92. 10.1016/j.molimm.2004.03.009 15140579

[50] Katoh Y Imakagura H Futatsumori M Nakayama K. Recruitment of clathrin onto endosomes by the Tom1-Tollip complex. Biochem Biophys Res Commun (2006) 341(1):143–9. 10.1016/j.bbrc.2005.12.156 16412388

[51] Ankem G Mitra S Sun F Moreno AC Chutvirasakul B Azurmendi HF The C2 domain of Tollip, a toll-like receptor signalling regulator, exhibits broad preference for phosphoinositides. Biochem J (2011) 435(3):597–608. 10.1042/bj20102160 21294713

[52] Baker B Geng S Chen K Diao N Yuan R Xu X Alteration of lysosome fusion and low-grade inflammation mediated by super-low-dose endotoxin. J Biol Chem (2015) 290(10):6670–8. 10.1074/jbc.M114.611442 25586187 PMC4358298

[53] Stahelin RV. Time to fold: Tom1 uses new tricks to regulate lipid binding of Tollip. Structure (2015) 23(10):1781–2. 10.1016/j.str.2015.09.003 26445490

[54] Xiao S Brannon MK Zhao X Fread KI Ellena JF Bushweller JH Tom1 modulates binding of Tollip to phosphatidylinositol 3-phosphate via a coupled folding and binding mechanism. Structure (2015) 23(10):1910–20. 10.1016/j.str.2015.07.017 26320582

[55] Kang RS Daniels CM Francis SA Shih SC Salerno WJ Hicke L Solution structure of a CUE-ubiquitin complex reveals a conserved mode of ubiquitin binding. Cell (2003) 113(5):621–30. 10.1016/S0092-8674(03)00362-3 12787503

[56] Mitra S Traughber CA Brannon MK Gomez S Capelluto DG. Ubiquitin interacts with the Tollip C2 and CUE domains and inhibits binding of Tollip to phosphoinositides. J Biol Chem (2013) 288(36):25780–91. 10.1074/jbc.M113.484170 23880770 PMC3764785

[57] Suetsugu S Kurisu S Takenawa T. Dynamic shaping of cellular membranes by phospholipids and membrane-deforming proteins. Physiol Rev (2014) 94(4):1219–48. 10.1152/physrev.00040.2013 25287863

[58] Piao W Song C Chen H Diaz MA Wahl LM Fitzgerald KA Endotoxin tolerance dysregulates MyD88- and toll/IL-1R domain-containing adapter inducing IFN-beta-dependent pathways and increases expression of negative regulators of TLR signaling. J Leukoc Biol (2009) 86(4):863–75. 10.1189/jlb.0309189 19656901 PMC2796624

[59] Diao N Zhang Y Chen K Yuan R Lee C Geng S Deficiency in toll-interacting protein (Tollip) skews inflamed yet incompetent innate leukocytes in vivo during DSS-induced septic colitis. Sci Rep (2016) 6:34672. 10.1038/srep34672 27703259 PMC5050405

[60] Mayer A Scheglmann D Dove S Glatz A Wickner W Haas A. Phosphatidylinositol 4,5-bisphosphate regulates two steps of homotypic vacuole fusion. Mol Biol Cell (2000) 11(3):807–17. 10.1091/mbc.11.3.807 10712501 PMC14812

[61] Devereaux K Dall’Armi C Alcazar-Roman A Ogasawara Y Zhou X Wang F Regulation of mammalian autophagy by class II and III PI 3-kinases through PI3P synthesis. PLoS One (2013) 8(10):e76405. 10.1371/journal.pone.0076405 24098492 PMC3789715

[62] Deretic V Saitoh T Akira S. Autophagy in infection, inflammation and immunity. Nat Rev Immunol (2013) 13(10):722–37. 10.1038/nri3532 24064518 PMC5340150

[63] Settembre C Fraldi A Medina DL Ballabio A. Signals from the lysosome: a control centre for cellular clearance and energy metabolism. Nat Rev Mol Cell Biol (2013) 14(5):283–96. 10.1038/nrm3565 23609508 PMC4387238

[64] Hsu HY Wen MH. Lipopolysaccharide-mediated reactive oxygen species and signal transduction in the regulation of interleukin-1 gene expression. J Biol Chem (2002) 277(25):22131–9. 10.1074/jbc.M111883200 11940570

[65] Keqiang C Ruoxi Y Yao Z Shuo G Liwu L. Tollip deficiency alters atherosclerosis and steatosis by disrupting lipophagy. J Am Heart Assoc (2017) 6(4):e004078. 10.1161/JAHA.116.004078 28396568 PMC5532987

[66] Li M Feng B Wang L Guo S Zhang P Gong J Tollip is a critical mediator of cerebral ischaemia-reperfusion injury. J Pathol (2015) 237(2):249–62. 10.1002/path.4565 26011492

[67] Steenholdt C Andresen L Pedersen G Hansen A Brynskov J. Expression and function of toll-like receptor 8 and Tollip in colonic epithelial cells from patients with inflammatory bowel disease. Scand J Gastroenterol (2009) 44(2):195–204. 10.1080/00365520802495529 18985539

[68] Maillard MH Bega H Uhlig HH Barnich N Grandjean T Chamaillard M Toll-interacting protein modulates colitis susceptibility in mice. Inflamm Bowel Dis (2014) 20(4):660–70. 10.1097/mib.0000000000000006 24572204

[69] Fernandes P MacSharry J Darby T Fanning A Shanahan F Houston A Differential expression of key regulators of toll-like receptors in ulcerative colitis and Crohn’s disease: a role for Tollip and peroxisome proliferator-activated receptor gamma? Clin Exp Immunol (2016) 183(3):358–68. 10.1111/cei.12732 26462859 PMC4750602

[70] Noth I Zhang Y Ma S-F Flores C Barber M Huang Y Genetic variants associated with idiopathic pulmonary fibrosis susceptibility and mortality: a genome-wide association study. Lancet Respir Med (2013) 1(4):309–17. 10.1016/S2213-2600(13)70045-6 24429156 PMC3894577

[71] Raghu G Collard HR Egan JJ Martinez FJ Behr J Brown KK An official ATS/ERS/JRS/ALAT statement: idiopathic pulmonary fibrosis: evidence-based guidelines for diagnosis and management. Am J Respir Crit Care Med (2011) 183(6):788–824. 10.1164/rccm.2009-040GL 21471066 PMC5450933

[72] Oldham JM Ma S-F Martinez FJ Anstrom KJ Raghu G Schwartz DA TOLLIP, MUC5B, and the response to N-acetylcysteine among individuals with idiopathic pulmonary fibrosis. Am J Respir Crit Care Med (2015) 192(12):1475–82. 10.1164/rccm.201505-1010OC 26331942 PMC4731723

[73] Cantu E Suzuki Y Diamond JM Ellis J Tiwari J Beduhn B Protein quantitative trait loci analysis identifies genetic variation in the innate immune regulator Tollip in post lung transplant primary graft dysfunction risk. Am J Transplant (2016) 16(3):833–40. 10.1111/ajt.13525 26663441 PMC4767612

